# Tip-sample interactions on graphite studied using the wavelet transform

**DOI:** 10.3762/bjnano.1.21

**Published:** 2010-12-22

**Authors:** Giovanna Malegori, Gabriele Ferrini

**Affiliations:** 1 Dipartimento di Matematica e Fisica, Università Cattolica del Sacro Cuore, I-25121 Brescia, Italy; 2 Dipartimento di Fisica, Università degli Studi di Milano, I-20122 Milano, Italy

**Keywords:** AFM, force, graphite, thermal excitation, wavelet transforms

## Abstract

Wavelet transform analysis is applied to a thermally excited cantilever to get insights into fundamental thermodynamical properties of its motion. The shortcomings of the widely used Fourier analysis are briefly discussed to put into perspective the wavelet transform analysis, used to describe the temporal evolution of the spectral content of the thermal oscillations of a cantilever with an interacting tip. This analysis allows to retrieve the force gradients, the forces and the Hamaker constant in a measurement time of less than 40 ms.

## Introduction

The non-contact atomic force microscopy (NC-AFM) is a powerful tool to study not only the surface topography, but also the mechanical and chemical characteristics of the sample at the nanoscale [1–3]. The tip of an excited cantilever is sensitive to both forces and force gradients, when approaching the sample surface. The response of the cantilever may show a modification of the oscillation amplitude, frequency, phase or damping. The measurement of these cantilever parameters allows to gain information on the physical properties of the sample with (sub-)molecular resolution [4–5]. The dynamic behavior of a weakly interacting cantilever vibrating near a resonance can be well approximated by a simple harmonic oscillator model, described by three independent parameters, resonance frequency, ω_0_, amplitude at resonance, *A*_0_, and quality factor, Q. A shift in ω_0_ is related primarily to the tip-surface force gradient, *A*_0_ to the driving force, and Q to the energy dissipation [2,6].

The thermal motion (or Brownian motion) of the cantilever’s tip is connected to the local mechanical compliance via the fluctuation-dissipation theorem. The cantilever thermal fluctuations are modified by the tip-surface interaction forces: monitoring these modifications allows to reconstruct the interaction potential and obtain information on various kinds of surface forces [7–9]. The influence of the local environment on the cantilever oscillations around the equilibrium position, detected by a quadrant photodiode in the optical beam deflection method, is usually analyzed by the Fourier transform, that represents the temporal fluctuations of the cantilever in the frequency domain. By doing so, the oscillation eigenmodes of the cantilever are displayed in the spectrum as resonance peaks. However, Fourier transform (FT) analysis is correctly interpreted (and useful) only in the case of stationary systems, i.e., the frequency spectrum must be correlated with a temporally invariant physical system. If the physical state of the system changes in time, the Fourier spectrum only displays an average of spectra corresponding to different states and so the physical information is no more correlated with a single state of the system.

There exists a powerful and well developed mathematical tool overcoming these limitations, not yet applied to analyze the dynamic force spectroscopy (DFS) data, the wavelet analysis [10–11]. In this work, we present wavelet theory as an advanced tool for the analysis and characterization of temporal traces obtained by DFS. A necessary mathematical background on wavelet theory is briefly introduced in the following sections, regarding specifically the decomposition of a one dimensional signal into its frequency components by scaled wavelet functions, known as continuous wavelet transform (CWT). Since wavelet functions are scaled according to frequency and time, such a decomposition results in the so-called *time-frequency localization*. The wavelet transform approach gives a meaningful and intuitive representation of the temporal evolution of the spectral content of an oscillating cantilever. CWT converts a one-dimensional time signal into a two dimensional time-frequency representation, which displays the signal amplitude localized in time and frequency on a time-frequency plane. This is particularly useful to study transitory regimes, i.e., signal with a frequency spectrum changing during the data collection. This work will show that the tip-sample interaction forces can be quantitatively measured using CWT with acquisition times as short as few tens of milliseconds, as required for practical DFS imaging.

Since wavelets are a mathematical tool, they have been used in a number of application in different fields of science and technology to extract information from and/or denoise many different kinds of data, including – but certainly not limited to – audio signals, images, optical spectra, time series. Previously, wavelet analysis has been used in atomic force spectroscopy mainly to denoise or extract data from images [12–13], which is by far the most important application of the wavelet transform.

In the following, first we briefly illustrate the Fourier approach to analyze the time traces of the cantilever thermal oscillations collected at different separations from the surface. Successively the CWT and its use in DFS will be introduced.

### Fourier analysis of the cantilever thermal fluctuations

**Figure 1:**
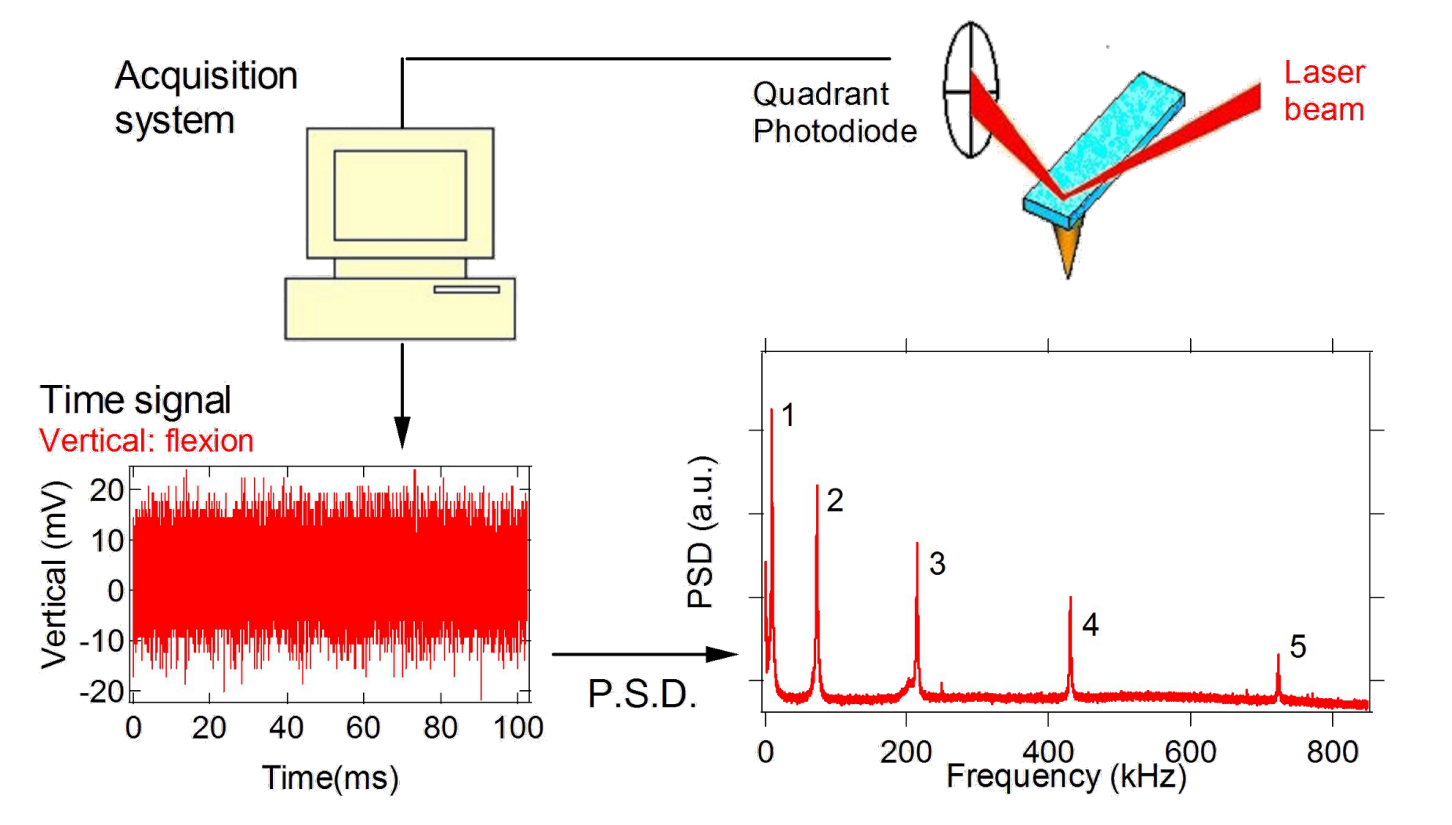
Block diagram of the optical beam detection system. A typical power spectral density spectrum of the cantilever flexural modes (up to the fifth) is shown.

Fourier analysis can be used to process the temporal trace of the cantilever thermal vibrations detected by a standard AFM optical beam deflection system. The power spectral density (PSD) of the time signal, extending over a temporal interval sufficiently long to assure the needed spectral resolution, reveals resonance peaks corresponding to the various oscillation eigenmodes of the cantilever beam (Figure 1). This analysis is repeated at various separations from the surface, up to the jump-to-contact distance. The force gradient of the interaction *dF*_ts_/*dz* (where F_ts_ is the tip-sample force and *z* the tip-sample distance, positive along the surface normal direction) is directly evaluated by the observed frequency shift of the PSD as a function of *z*. Considering each flexural mode equivalent to a mass-spring system, the tip-sample interaction elastic constant *k*_ts_ = −*dF*_ts_/*dz* is expressed as a function of the resonant frequency as 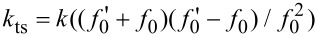, where 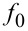 is the resonant frequency of the free cantilever, 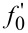 is the resonant frequency of the cantilever interacting with the surface force gradients and *k* is the equivalent elastic constant of the mode under consideration. This relation holds if *k*_ts_ remains constant for the whole range of the displacements from the equilibrium position covered by the cantilever. This is usually true in the thermal regime since we are dealing with small oscillations (less than 0.2 nm) [9]. If 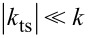 the frequency shift 
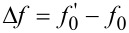
 is proportional to the interaction elastic constant 
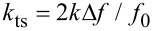
 [1].

From the same PSD, besides the force gradient, it is possible to measure the quality factor *Q* of the mode, that is determined by the relative width of the resonance peaks corresponding to the oscillation eigenmodes of the cantilever (*Q* = Δω/ω_0_). *Q* is usually dependent on the distance from the surface. Since the quality factor *Q* is connected to dissipation, important informations on the tip-sample energy exchange can be retrieved.

**Figure 2:**
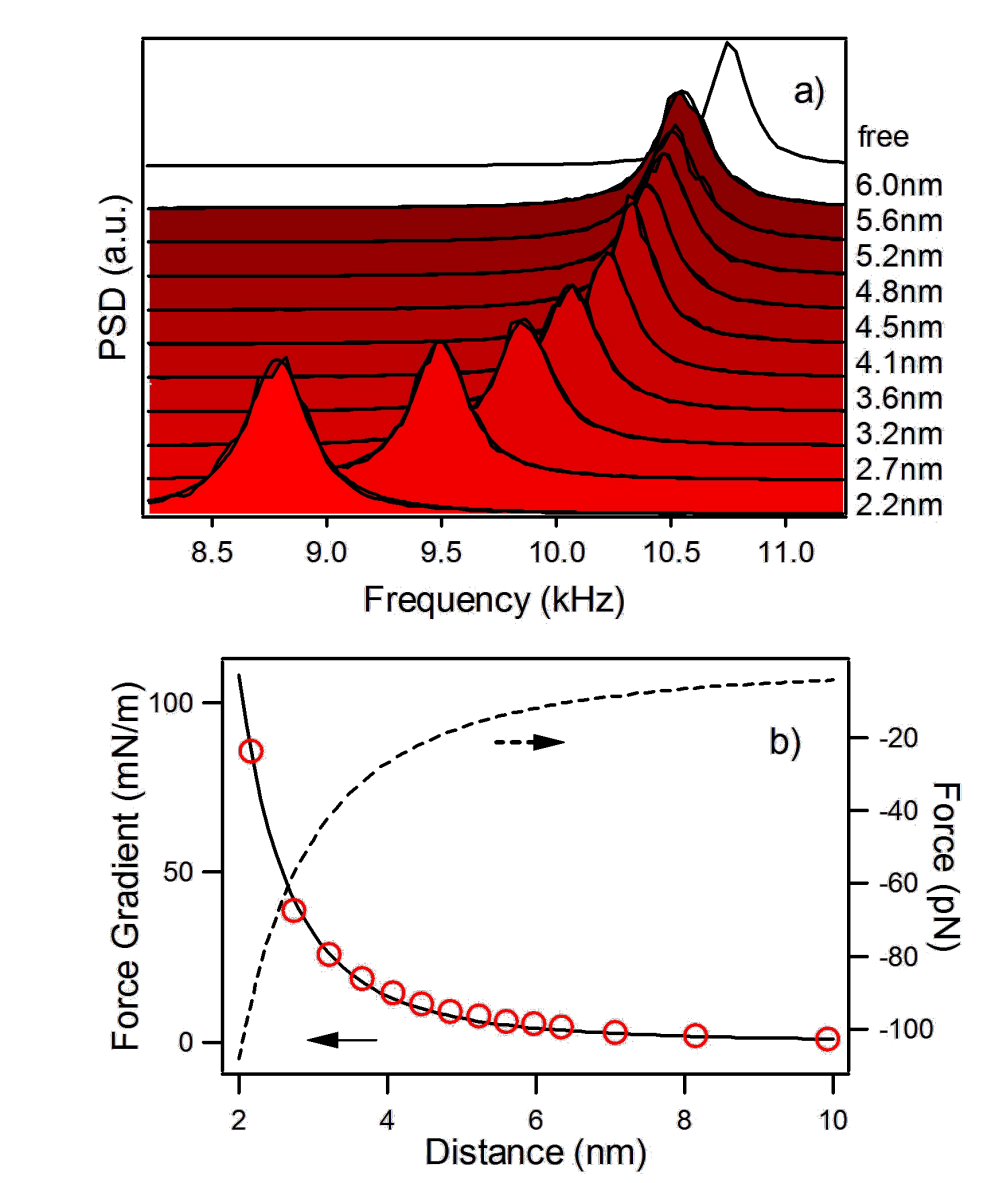
Results from the Fourier transform method, adapted from [9]. a) Power spectral density of the thermal fluctuations of the first flexural mode of the cantilever acquired at different tip-sample separations. A negative frequency shift of the resonant frequency is observed on approaching the graphite surface. The resonance peaks are fitted with a Lorentzian. b) The black continuous line is a fit of the van der Waals force gradient between a spherical tip and a flat surface (force gradient = *HR*/3*z*^3^, *z* is the tip-sample distance) to the measured frequency shift of the first flexural mode as a function of the tip-surface separation (red circles). The dashed line is the interaction force obtained by integration.

With this techniques force gradients and quality factors on graphite in air have been measured [9]. It was found that the attractive force gradient data are well reproduced by a nonretarded van der Waals function in the form *HR/*(3*z*^3^) (*H* is the Hamaker constant and *R* the tip radius of curvature), up to the jump-to-contact distance *D* which occurs at around 2 nm from the surface (Figure 2). In this distance range, *Q* is almost constant for the first and second flexural modes. This means that the interaction is conservative at distances greater than *D*, the first flexural mode showing an evident decrease of the *Q* value just before the jumps-to-contact. The dissipation mechanism related to this sharp transition is due to a local interaction of the tip apex with the surface.

In these experiments, the acquisition and storage of the photodiode time signal requires tens of seconds at each tip-sample separation. This implies that the measurements at a single spatial location (one pixel of an image) may take minutes. The long measurements duration, besides the control of thermal drifts, is not practical for imaging pourposes.

In closing this section, it is interesting to note that near the sample, the quality factor is lower than that of the free cantilever. The decrease is due to the interaction of the rectangular beam with the sample surface. If the tip-sample separation is very small, the distance between the beam and the surface is about the tip height (nominal value *h* = 20–25 μm). When the cantilever oscillates in air or in a fluid close to a solid surface, due to a confinement effect, an increased damping is manifested as a decrease of the quality factor [14]. This effect is relevant for piezotube movements on the μm scale but not on the nm scale covered by the present measurements, where the effect of the tip-sample interaction dominates.

### Continuous wavelet transform and time-frequency resolution

The FT analysis provides a frequency representation of a signal with perfect spectral resolution but without the possibility to correlate the frequency spectrum with the signal evolution in time. Instead, a time-frequency representation shows the signal evolution over both time and frequency. CWT is a refined alternative to the classical windowed Fourier analysis, providing not only the representation of the spectral energy content of the signal at a certain time, but also the ability to adapt the resolution to the signal frequency.

A wavelet is a smooth function Ψ(*t*) with a compact support (or a rapid decay at infinity, contrary to the Fourier basis), and zero average,


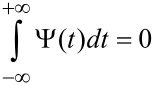


which is translated in time by *d* and dilated by a positive scale parameter *s*,


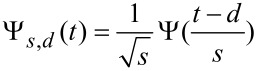


The zero average condition imply that Ψ(*t*) is an oscillating function. The function Ψ(*t*) is called a mother wavelet, the translated and dilated replicas Ψ*_s,d_*(*t*) are called daughter wavelets. The wavelet transform of a function of time *t*, *f*(*t*), at the scale *s* and delay *d* is computed by correlating *f*(*t*) with the daughter wavelet at the corresponding scale and delay,


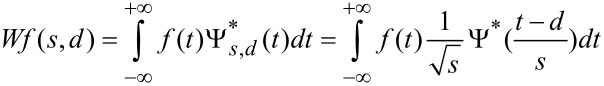


**Figure 3:**
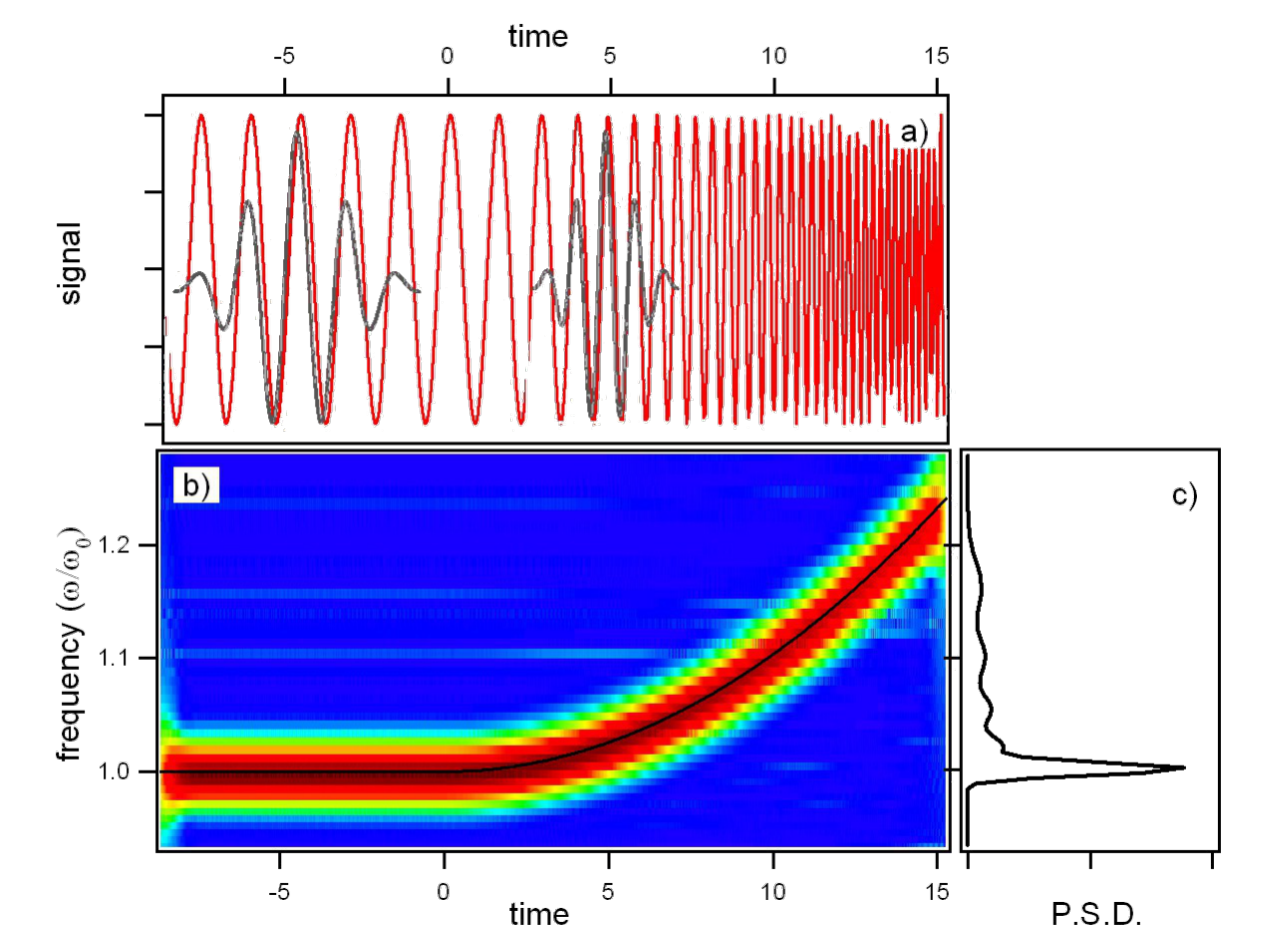
Comparison between the Fourier transform and the wavelet transform analysis. a) The time signal, a cosine function for negative times and a cosine with quadratic chirp for positive times. Two daughter wavelet functions with different dilations and delays are superposed to the signal to show the local resemblance between signal and wavelet. b) Wavelet transform of the temporal trace represented in a) showing the evolution of the signal frequency. The black line is the calculated instantaneous frequency. c) Fourier transform (power spectral density) of the signal represented in a). Only an average of the signal frequencies is observed.

The wavelet transform coefficients *Wf*(*s,d*) are “resemblance” coefficients, that measure the similitude between the signal and the wavelet atoms at various scales and delays (Figure 3a).

The square modulus of the wavelet coefficients |*Wf*(*s,d*)|^2^ is proportional to the local energy density of the signal at the given delay and scale, called the scalogram of the signal. As explained in detail below, the delay-scale representation in which wavelets are defined can be mapped into the more physical time-frequency representation to describe the signal energy localization in frequency and time. It is useful to point out that the instantaneous frequency of the signal can be traced by the so called wavelet ridges analysis of the spectrogram in the time-frequency plane. The wavelet ridges are the maxima points of the normalized scalogram [11], showing the instantaneous frequencies within the limits of the transform’s resolution (the ridge analysis will be useful to represent the experimental data). When the signal contains several spectral lines whose frequencies are sufficiently apart, the wavelet ridges (i.e., the local maxima) separates each of these components during their temporal evolution, a task that cannot be performed using Fourier analysis.

To visualize the differences between the FT and CWT consider a signal *f*(*t*) = *a*cosφ(*t*) with time varying phase φ(*t*), where φ(*t*) = ω_0_*t* at negative times and φ(*t*) = ω_0_*t* +α*t*^3^ at positive times (Figure 3a). The instantaneous pulsation is the derivative of the phase ω(*t*) = φ'(*t*) (the black line in Figure 3b). Since FT is a time invariant operator, only an average of the time dependent spectrum is observed (Figure 3c). On the other hand, CWT approach combines the time domain and frequency domain analysis so that the evolution of each spectral component is determined. The wavelet analysis allows to extract accurately the instantaneous frequency information even for rapidly varying time series (Figure 3b).

In the remainder of this section, we highligth the main features of CWT analysis that are important when applied to the time evolution of the cantilever oscillations. Unlike FT, the basis of CWT is not unique, so it is important the choice of the wavelet basis. In this work, we use a complex mother wavelet (also called the Gabor wavelet or the Gaussian wavelet) represented as


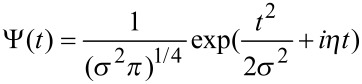


where σ controls the amplitude of the Gaussian envelope, and thus its time/frequency resolution, η the carrier frequency. Since the intrinsic time-frequency resolution in CWT is set by the atoms over which the signal is expanded, we chose this wavelet because it is particularly adapted to follow signals in time, having the least spread in both frequency and time domain and thus the best time frequency resolution.

The CWT is defined in terms of delays and scales and, as anticipated, the representation can be mapped to time and frequency. While it is immediate the connection of delay to time, some comments are useful to connect scale to frequency.

The signal relative to the vertical cantilever displacement, recorded with a digitizing oscilloscope from the optical beam deflection system photodiode, can be thought as a one dimensional string of sampling units. Each sampling unit is the value of the signal at a specific sampling time and together constitute the discretized sampled signal. A sampling unit is temporally connected to the next by a (usually) fixed sampling interval *T*. In this framework, the temporal parameter *t* in the expression of the Gabor wavelet can be regarded as a (adimensional) discrete index and likewise σ and η are adimensional wavelet parameters defining the wavelet shape over the discrete sampling string. The Gabor wavelet (adimensional) center frequency at scale *s* is given by *f* = η/(2π*s*). It is possible to associate a pseudo frequency *F* (in Hz) at a scale *s* by considering that *f* is sampled with a time interval *T*, so that *F* = *f/T*. Therefore, the wavelet dilations set by the scale parameter *s* are inversely proportional to the frequency *F*.

**Figure 4:**
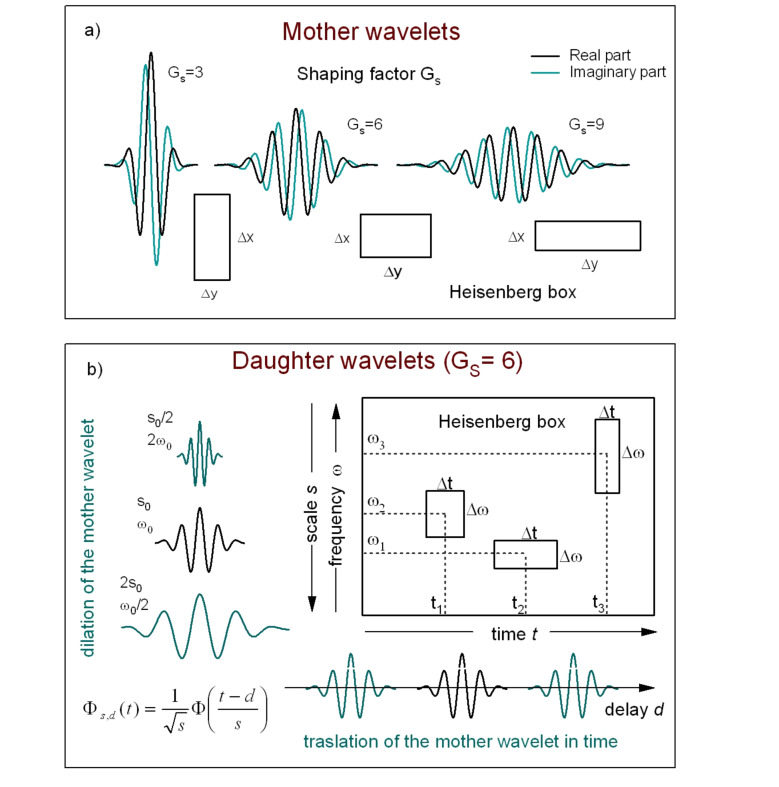
a) Complex Gabor wavelet with different shaping factors. An increase of *G*_S_ corresponds to more oscillations under the envelope. The "Heisenberg box" shows the relationship between the time and frequency resolution, like the uncertainty principle in quantum mechanics (adapted from [15]). b) A graphical representation of the delay and dilation transformations used in the continuous wavelet transform (adapted from [16]).

Strictly connected to the relation between scale and frequency is the wavelet time-frequency resolution. The joint time and frequency limitations set to the analysis of the energy content of the signal leads naturally to the introduction of the Heisenberg box, associated to each analyzing wavelet. The Heisenberg box delimits an area in the time-frequency plane over which different CWT coefficients cannot be separated, providing a geometrical representation of the Heisenberg uncertainty principle (Figure 4). We adopt the commonly used definition of the measure of the uncertainty window Δ as the root-mean-square extension of the wavelet in the corresponding time or frequency space,


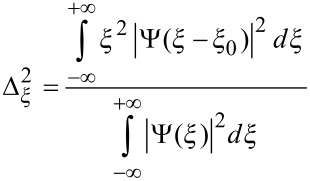


where ξ_0_ is a translation parameter and Ψ(ξ) represents the Gabor mother wavelet, expressed either in time, ξ = *t*, or circular frequency, ξ = ω = 2π*F*, Ψ(ω) = FT(Ψ(*t*)).

The time-frequency resolution of the analyzing Gabor mother wavelet, used in this work, is determined by the σ parameter. The Heisenberg box associated to the mother Gabor wavelet is given by a time resolution 
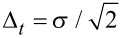
 and a frequency (or pulsation) resolution 
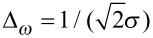
. When the wavelet is subject to a scale dilatation *s*, the corresponding resolution has size Δ*_s,t_* = *s*Δ*_t_* along time and Δ*_s,ω_* = Δω/*s* along frequency (Figure 4). The Heisenberg box centered at time *t* and frequency ω = 2π*F* is thus defined as


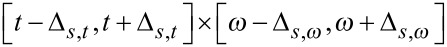


As expected from the uncertainty principle, Δ*_s,t_*Δ*_s_*_,ω_ = 1/2.

**Figure 5:**
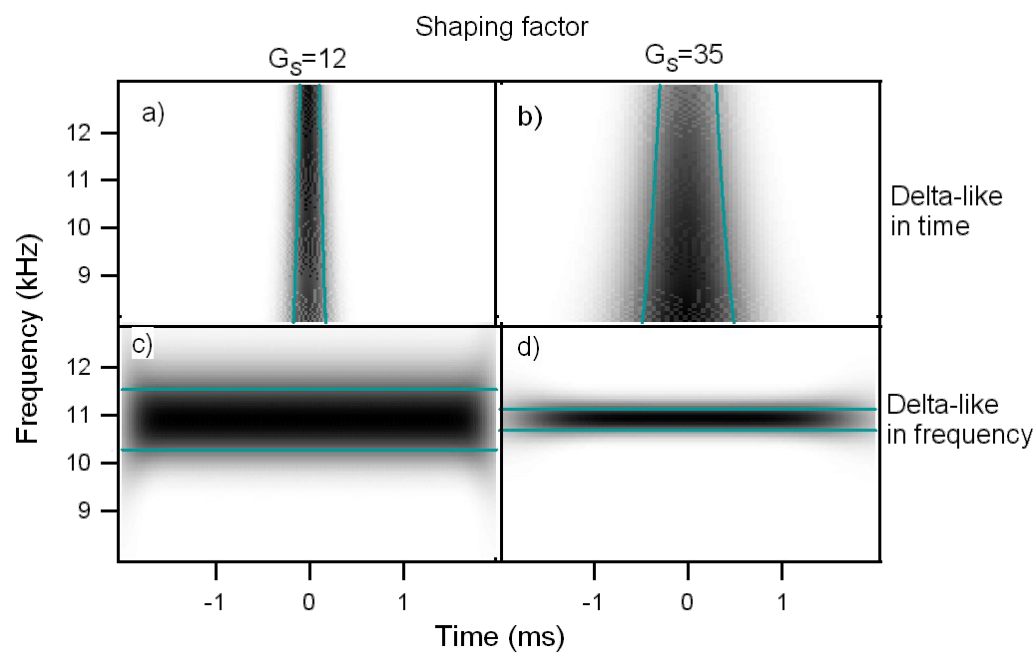
Continuous wavelet transform of a delta-like signal in time and a delta-like signal in frequency, analyzed with wavelets of different shaping factor, a-c) *G*_S_ = 12, b-d) *G*_S_ = 35. a-b) CWT of a delta-like function in time. The dependence of resolution on scale (frequency) is clearly shown. c-d) CWT of a delta-like function in frequency. The frequency resolution increases with the shaping factor. The degradation of the CWT resolution near the edges of the window transform is visible (edge effect).

It is useful to define the dimensionless parameter known as the Gabor shaping factor *G*_S_ = ση [16], which takes in to account the envelope width (temporal resolution) and the number of oscillations within the envelope width (frequency resolution). The shaping factor controls the time frequency resolution via the dimensions of the Heisenberg box (Figure 4). In fact, as it is easily seen, 
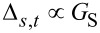
 while 
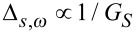
, so that the choice of the single parameter *G*_S_ determines the shape of the Heisenberg box. An increase of *G*_S_ means more oscillations under the wavelet envelope and a larger time spread, the frequency resolution being improved and the time resolution degraded. In Figure 5 are shown the CWT of delta-like signals in time and frequency, whose time-frequency resolution is due only to the wavelet analyzing characteristics. As discussed above, it is possible to see that the frequency resolution due to the mother wavelet choice increases with *G*_S_ while the temporal resolution is degraded. The delta-like signals in time show clearly that the time resolution depends on the scale (frequency) parameter, increasing at lower scale (higher frequency). The delta-like signals in frequency also show the edge effect, a degradation of the wavelet resolution near the edges of the CWT time window due to the spectral broadening produced by the signal truncation.

## Results and Discussion

### Wavelet analysis of the cantilever thermal fluctuations

**Figure 6:**
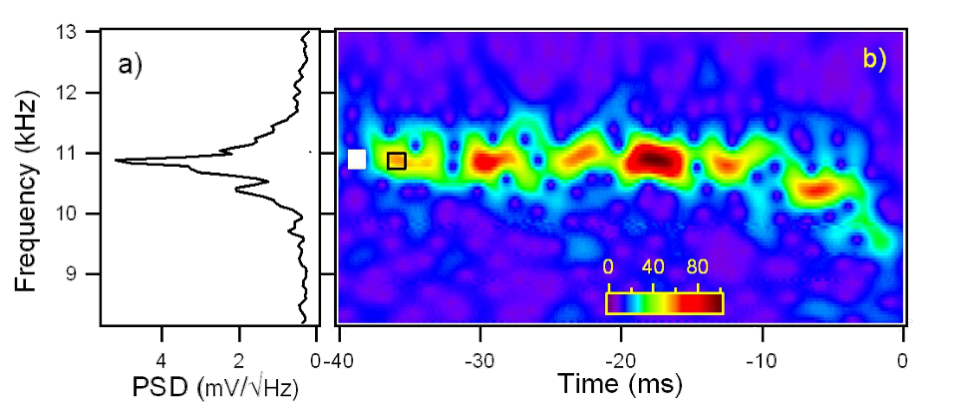
a) Power spectral density of the Brownian motion of the first flexural mode of the same temporal trace used for the wavelet transform on the right. b) Wavelet transform of the Brownian motion of the cantilever first flexural resonance, around its instantaneous equilibrium position, as the tip approaches the surface at constant velocity (9 nm / 40 ms = 225 nm/s). The wavelet coefficients |*Wf*(*f,t*)| are coded in colorscale. The origin of the time axis corresponds to the instant when the jump-to-contact occurs. The white box at the left side represents the Heisenberg box, the open box delimited by black lines represents the damped oscillator in response to an impulsive thermal excitation.

The wavelet analysis is applied to the force–distance curves taken with the cantilever subject to thermal fluctuations while approaching the surface. Figure 6 shows the scalogram of a 40 ms sampling of the cantilever Brownian motion around its instantaneous equilibrium position while the piezo scanner is displaced at constant velocity to move the tip towards the surface, until it jumps to contact.

The discontinuous appearance of the signal in the time-frequency representation is due to the statistical nature of the cantilever excitation. The thermal contact of the cantilever with a reservoir at temperature *T* implies that its mean potential energy 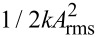 (where *A*_rms_ is the root mean square cantilever displacement due to thermal motion) is equal to 1/2*k*_B_*T* by the equipartition theorem, where *k*_B_ is the Boltzmann constant and *T* is the temperature. Microscopically this can be regarded as the action of random thermal kicks (i.e. uncorrelated impulsive forces), a driving force with white frequency spectrum. This thermal force induces cantilever displacements from the equilibrium position, that show a marked amplitude enhancement in correspondence of the flexural eigenfrequencies. Since the cantilever is subjected also to dissipative friction forces, the amplitude response of the cantilever around a flexural resonant frequency is not delta-like, but has a finite linewidth. The PSD of the same temporal trace used for the CWT, reported in Figure 6a, shows a linewidth comparable to the frequency indetermination of the Heisenberg box of the CWT and a structure at low frequency that is reminescent of the interaction with the surface, when for a short time the cantilever frequency is lowered.

It is interesting to clarify the origin of the “bumps” observed in the time-frequency representation. When the cantilever has a thermally activated fluctuation, each flexural mode responds as a damped harmonic oscillator whose equation of motion is 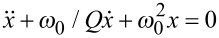 where *x* is the oscillation amplitude, *Q* the quality factor and ω_0_ the resonance frequency [17–18]. Considering for simplicity the initial conditions *x*(0) = *x*_0_, 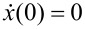 and assuming *Q* 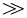 1, the solution is an exponentially decaying amplitude oscillating at the resonance frequency: 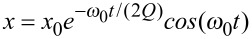.

The energy associated to the oscillator *E*(*t*) is proportional to 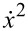 and from the above relations we see that the associated exponential energy decay time is τ = *Q/*ω_0_. The spectral energy density of the damped oscillator (*L*(ω)) is proportional to the square modulus of the Fourier transform of *x*(*t*), *L*(ω) = |FT(*x*(*t*))|^2^. Under the assumption *Q* 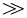 1, *L*(ω) is well approximated by a Lorentzian with a full width at half maximum of Δω = 2πΔ*f* = 1/τ.

Since the cantilever is first thermally excited and then damped to steady state by random forces that act on a much smaller time scale than its oscillation period, the characteristic response time for an isolated excitation/decay event cannot be smaller than 2τ, with an associated Lorentzian full width at half maximum of Δω.

From the above reasoning, it is natural to introduce the *damped oscillator box*, a geometrical representation of the extension in the time-frequency plane of the wavelet coefficients associated to a single excitation/decay event, centered at time *t* and frequency ω, defined as


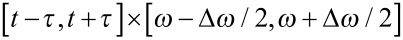


The damped oscillator box, contrary to the Heisenberg box, does not represent a limitation in resolution due to the wavelet choice, but a physical representation of the damped oscillator time frequency characteristics. It is important to note that the ultimate resolution limitations imposed by the Heisenberg box associated with the analyzing wavelet could prevent the observation of the true dimensions of the damped oscillator box.

Due to their different definitions, a comment on the sizes of the Heisenberg box and the damped oscillator box is useful. The Heisenberg box dimensions are the root-mean-square extensions of the Gabor wavelet envelope (i.e., its modulus) in time and frequency. Since the Gabor wavelet evelope is a gaussian in time and frequency, its root-mean-square extension is by definition the gaussian standard deviation, i.e. the half width at 
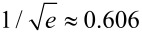
 of the maximum. The damped oscillator box dimension in frequency is the full width at half maximum of *L*(ω). In terms of the wavelet envelope (proportional to 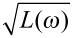), it is the full width at 
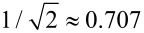
 of the maximum. The damped oscillator box dimension in time is 2τ, where τ is the full width at 
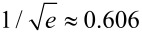
 of the maximum of the exponentially decaying oscillator amplitude.

**Figure 7:**
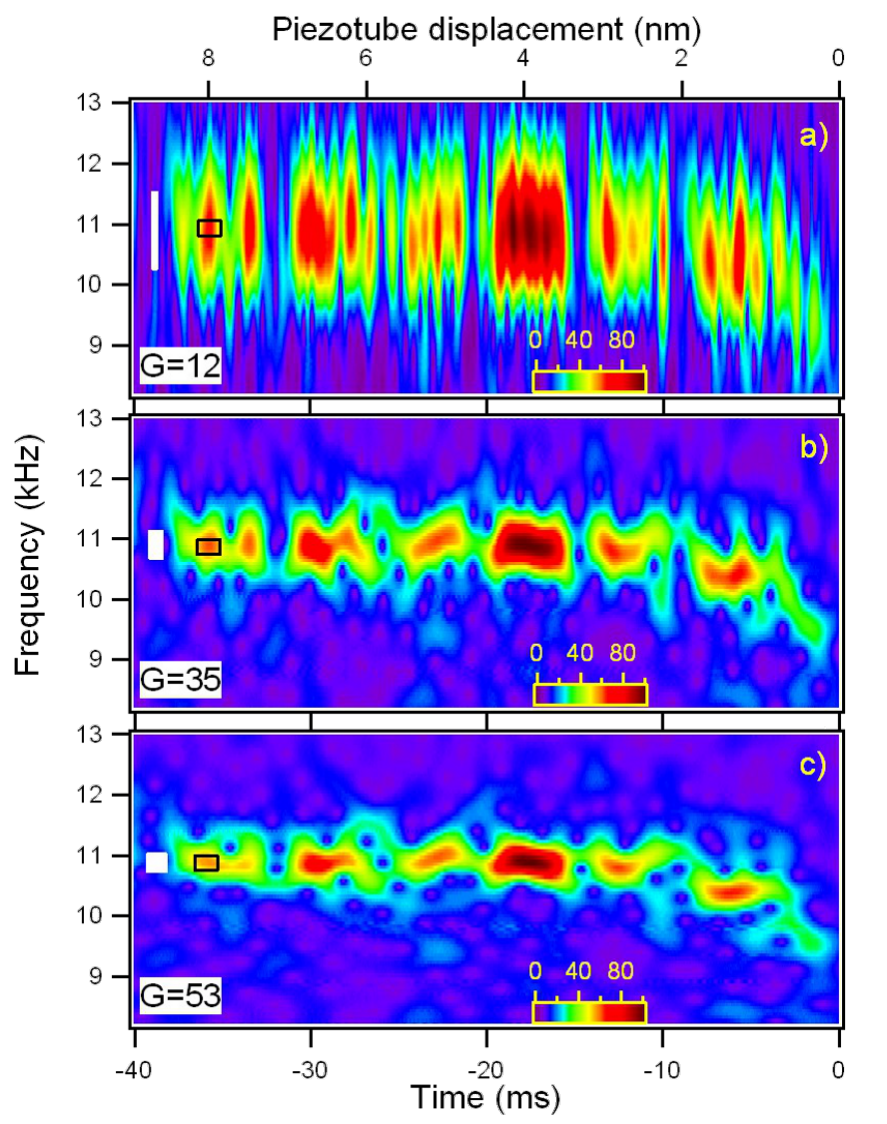
Wavelet transform of the cantilever thermal fluctuations around its instantaneous equilibrium position, using three mother wavelet with different shaping factor, a) *G*_S_=12, b) *G*_S_=35, c) *G*_S_=53. Increasing the shaping factor improves the frequency resolution but lowers the time resolution. The tip is moved toward the surface at a velocity of ≈225 nm/s until it jumps to contact (corresponding to the origin of the time axis). The wavelet coefficients |*Wf*(*f,t*)| are coded in colorscale. The white boxes at the left sides are the Heisenberg boxes. The open boxes delimited by black lines represent the damped oscillator boxes.

We did not attempt to correct the sizes of the boxes using a single common definition because the comparisons with the experimental data in the present work are mainly qualitative. In our case *Q* = 43 and *f*_0_ = 10.9 kHz for the first flexural mode, implying τ = 1.25 ms and Δω = 250 Hz. It is important to note that the temporal and frequency width of many discrete time frequency small structures seen in the CWT of the cantilever thermal signal in Figure 7c are of the same dimensions of the damped oscillator box 2τ × Δω. This observation is possible because the first flexural mode is represented with a Gabor wavelet with a shaping factor *G*_S_ = 53 around the resonant frequency, the Heisenberg box (1.1 ms × 290 Hz) is similar to the damped oscillator box (1.25 ms × 250 Hz). In the representation of Figure 7a and Figure 7b, the CWT has different shaping factors and thus different dimensions of the Heisenberg box (0.71 ms × 450 Hz for *G*_S_ = 35, Figure 7b, 0.25 ms × 1300 Hz for *G*_S_ = 12, Figure 7a), that allows to measure the time width of the damped oscillator structures, but not its frequency width due to limited frequency resolution. It is important to note that the temporal width of the structures is independent on the time resolution of the wavelet, indicating that we are observing a real physical feature, that is not related to the choice of the wavelet representation. As a rule of thumb, CWT should allow to follow more easily the single-thermal-excitation-event time decay in high-*Q* environments and measure its frequency linewidth in low-*Q* environments.

**Figure 8:**
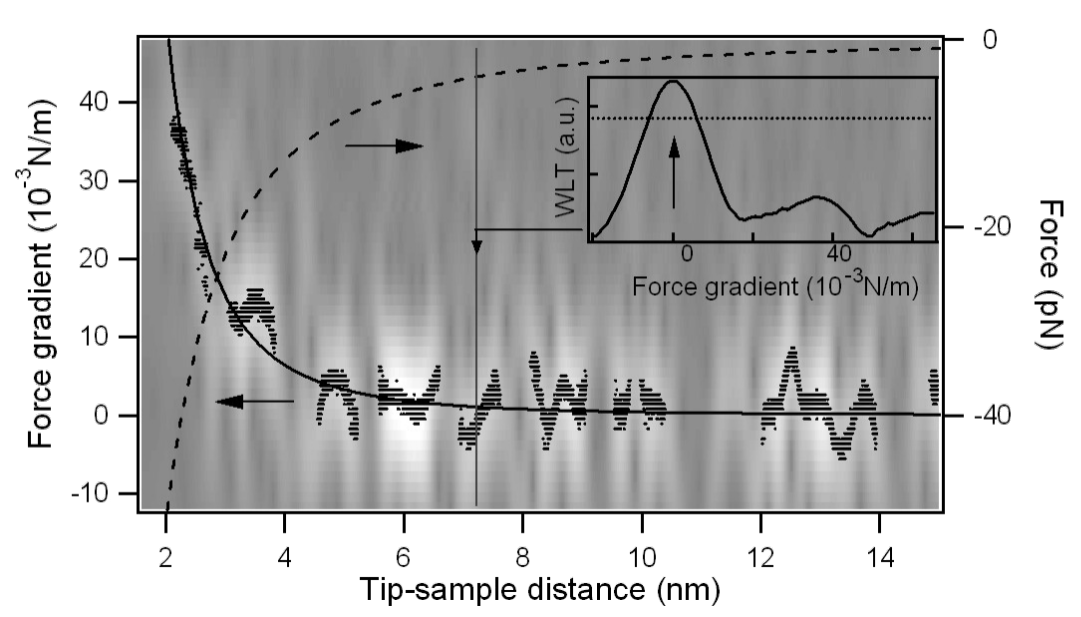
Force gradient versus tip-sample distance for the first flexural mode near the jump-to-contact. The wavelet ridges provide the instantaneous frequencies within the limits of the scalogram resolution. The wavelet ridges are the local maxima of the normalized scalogram above a specified threshold, as schematically shown in the inset. The threshold is represented by a horizontal line and the maximum point is indicated by an arrow for a vertical cut of the data at constant tip-sample distance. The CWT of Figure 7b is represented in gray scale on the background together with its ridges (black points). The continuous black line is an Hamaker-like force gradient function fitted to the wavelet ridges, the dashed line the force calculated by integration.

The first flexural mode frequency shift near the surface (Figure 7b) provides a complete force distance curve. The instantaneous frequency is evaluated by the wavelet ridges, the local maxima points of the normalized scalogram. In order to reduce noise effects, only maxima above a threshold are considered (see the schematic representation in the inset of Figure 8).

From the instantaneous frequency shift the gradient of the tip-sample interaction forces (*dF*_ts/_*dz*) is retrieved, using the relations previously reported, and the time scale is converted into the tip-sample separation by taking into account either the piezoscanner velocity and the cantilever static deflection, to obtain a complete force gradient versus distance curve (Figure 8). The gradient data from CWT ridges are well fitted by a nonretarded van der Waals function in the form *HR*/3*z^−^*^3^, with *HR* = 1.2 × 10^−27^ Jm. Using the typical values of *H* in graphite (*H* = 0.1 aJ), the tip radius is evaluated as *R* = 12 nm, in good agreement with the nominal radius of curvature given by the manufacturer (*R* = 10 nm). To promote this technique from proof of principle to a measurement of the Hamaker constant with a good lateral resolution, a thorough characterization of the tip radius of curvature is needed.

Finally, we note that the whole force curve is acquired in less than 40 ms, a time significantly less than that usually needed for force versus distance measurements. With an optimization of the electronics and reduction of dead times in the acquisition process, it would be possible to acquire images in which a complete information on force gradients and topography is compatible with 1–30 ms/pixel data acquisition times required for practical DFS imaging.

## Conclusion

The interaction of an AFM cantilever tip with a graphite sample is measured by applying the wavelet transform analysis to its Brownian motion near the surface. The wavelet transform analysis is a mathematical tool able to analyze the instantaneous spectral content of rapidly varying signals. Using the wavelet transforms to analyze the temporal traces of the thermal motion superposed on a force-distance curve, the tip-sample interaction is measured in tens of ms, a time compatible with imaging acquisition rates. The wavelet transform technique is very promising since the analysis could be applied simultaneously to the higher flexural eigenmodes. Moreover the measurement could be carried out across the jump-to-contact transition without interruption, providing information on the elastic response of the surface.

## Experimental

The experiments are carried out with an AFM [19] mounted on a massive platform suspended by springs to provide isolation from external mechanical noise. The AFM with its isolation platform are closed inside an acoustic isolation chamber. The cantilever deflection is monitored by an optical beam deflection system based on a 600 nm laser diode coupled to a monomode fiber (with a mode field diameter of 4 μm), which acts as a mode filter, giving a TEM_00_ beam output after recollimation. The collimated fiber output is focalized with an aspherical lens to a 10 μm spot on the cantilever end. A digitizing oscilloscope collects the differential outputs (left-right and top-bottom) of the four quadrant silicon diode. The overall bandwidth of the beam deflection system exceeds 1 MHz. The digitizing oscilloscope has a 8 bit vertical resolution, 250 MHz analog bandwidth, 1 Gsample/s maximum sampling rate, and a buffer memory of 128 Msample.

The silicon cantilevers average dimensions are 40 × 460 × 2 μm with a typical tip radius *R* = 10 nm. The resonance frequency of the first flexural mode of the cantilever used in the experiments is *f*_0_ = (10.908 ± 0.002) kHz, its elastic constant is *k* = 0.13 N/m [20]. For each cantilever the elastic constant is evaluated both by the Sader method [20] and the thermal noise method applied to the first flexural mode [21–22]. Both methods agree within 5%.

The piezoscanning system is based on a single scanner tube with a maximum vertical extension of 2 μm. The experiments consisted in acquiring the temporal evolution of the thermal noise as a function of the tip-sample distance. The thermal noise signal measured by the beam deflection system is sampled with the digitizing oscilloscope while the tip moves toward the surface. The piezoscanner is displaced at constant velocity of approximately 225 nm/s. The sampling time is 240 ns so that the signal string is composed by 4166 sampling points every ms of acquisition time. The CWT analysis is performed off-line.

The sample consisted of a freshly cleaved highly oriented pyrolitic graphite (HOPG) surface. All the experiments have been conducted in air, with a relative humidity of less than 50%. Figure 1 schematically shows the experimental apparatus: the electronic noise level is small enought to detect up to five flexural eigenmodes. The optical lever sensitivity is calibrated by taking the force spectroscopy curves on the hard HOPG surface, assuming a negligible indentation and thus equal distances spanned by the cantilever tip and the piezotube. The obtained sensitivity is in the range of 50–200 nm/V, depending on the cantilever type, beam position, and laser light power level. The cantilever has a 15° tilt with respect to the horizontal plane (that coincides with the sample surface), which is considered for sensitivity correction [23]. Since the laser beam position influences the effective length of the cantilever and the sensitivity, the stability of the laser alignment is carefully controlled during the measurements. From the approach force curves after the jump to contact, the tip-sample contact point is determined as the distance at which no force acts on the cantilever that is when the cantilever is not deflected.
